# Supplementary figures and images for: Learning unsupervised feature representations for single cell microscopy images with paired cell inpainting
Source: PLoS Comput Biol. 2019 Sep 3;15(9):e1007348. doi: 10.1371/journal.pcbi.1007348 (PMC6743779; doi:10.1371/journal.pcbi.1007348)

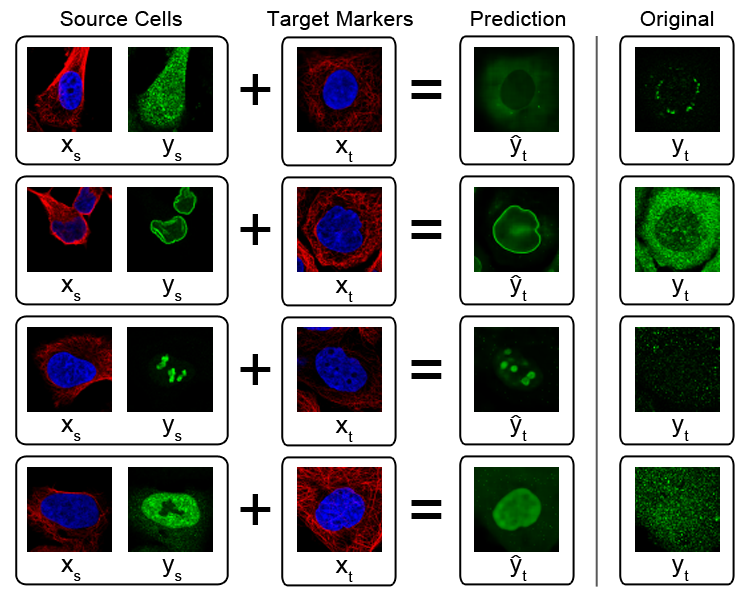

Supplement: S1 Fig — We take four source human cells with different localizations (left-most boxes), belonging in the cytosol, nuclear membrane, nucleoli, and nucleoplasm, from top to bottom. For each of our source cells, we run our human cell model end-to-end with target markers from different images (left-center boxes) as input to generate predictions of the protein localization in these target cells (right-center boxes). We show the actual protein localization of the target cells (right-most boxes) to demonstrate that our model is capable of synthesizing results based on the source cells even when there is a mismatch between the source and target cell protein localization. (TIF) [file pcbi.1007348.s001.tif]

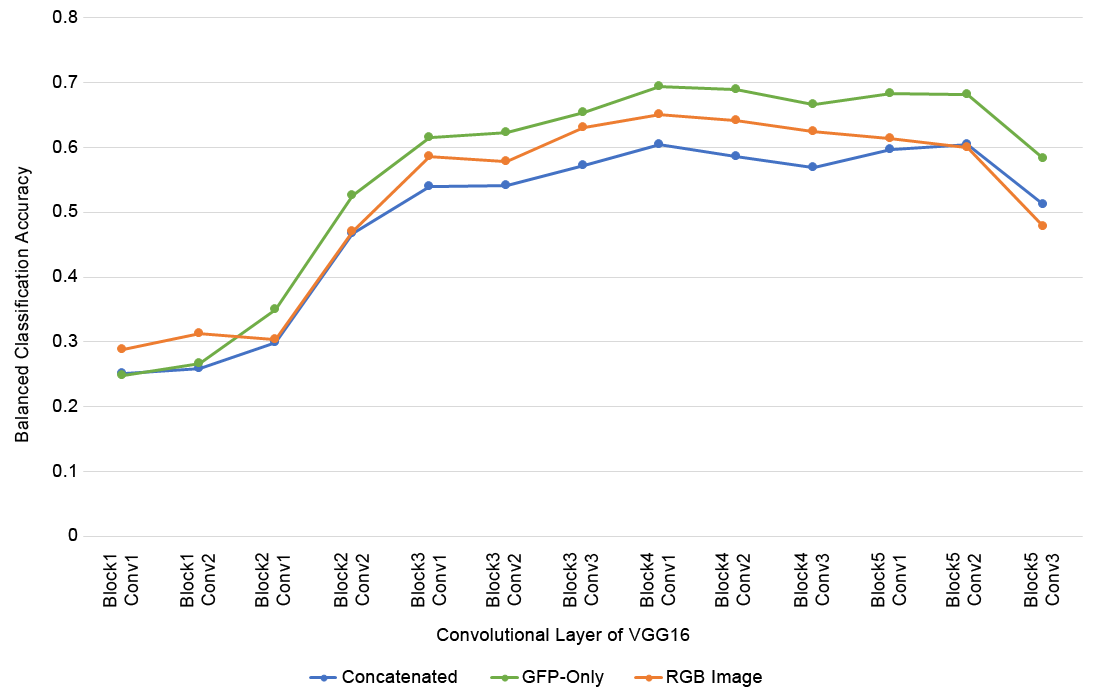

Supplement: S2 Fig — Images in the yeast single cell classification dataset were inputted using three different strategies: for “Concatenated”, we inputted the RFP and GFP channels independently as greyscale images and concatenated the representations; for “GFP-Only”, we inputted the GFP channel as a greyscale image and used this representation alone; and for “RGB Image”, we arbitrarily mapped channels to RGB channels (RFP to red, GFP to green, and blue left empty). Feature representations were extracted by maximum pooling the feature maps over spatial dimensions. We report the balanced classification accuracy using a leave-one-out kNN classifier (k = 11) for these representations, identical to the one described in the “Paired cell inpainting features discriminate protein subcellular localization in yeast single cells” section of the Results. (TIF) [file pcbi.1007348.s002.tif]

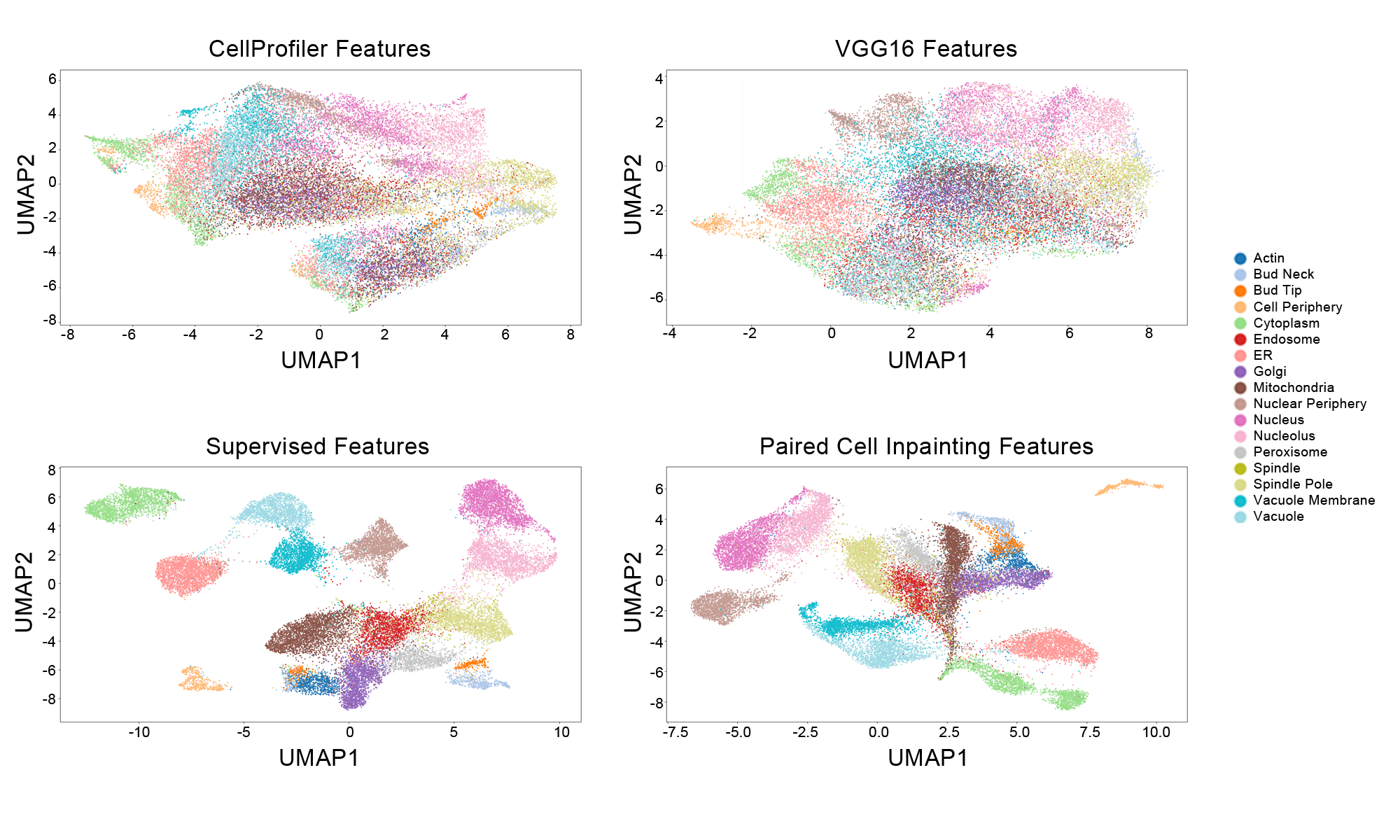

Supplement: S3 Fig — All UMAPs are generated with the same parameters (Euclidean distance, 30 neighbors, minimum distance of 0.3). Embedded points are visualized as a scatterplot and are colored according to their label, as shown in the legend to the right. (TIF) [file pcbi.1007348.s003.tif]

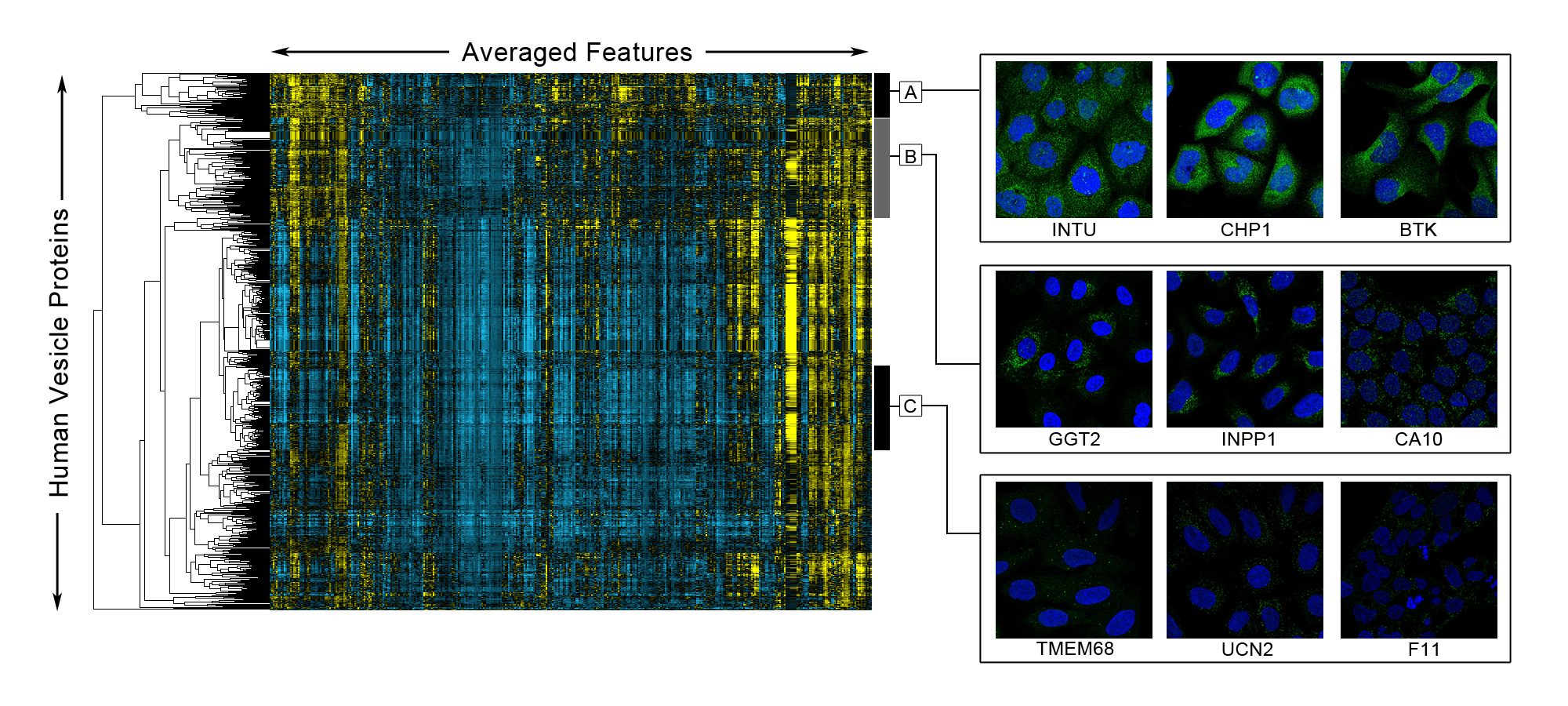

Supplement: S4 Fig — We visualize features as a heat map, where positive values are colored yellow and negative values are colored blue, with the intensity of the color corresponding to magnitude. Columns in this heat map are features, while rows are proteins. Features have been mean-centered and normalized using all proteins in the dataset. We show three clusters (black and grey bars on the right), and crops of three representative images of the proteins within each of the clusters. For image crops, we show the protein channel in green, and the nucleus channel in blue. (TIF) [file pcbi.1007348.s004.tif]

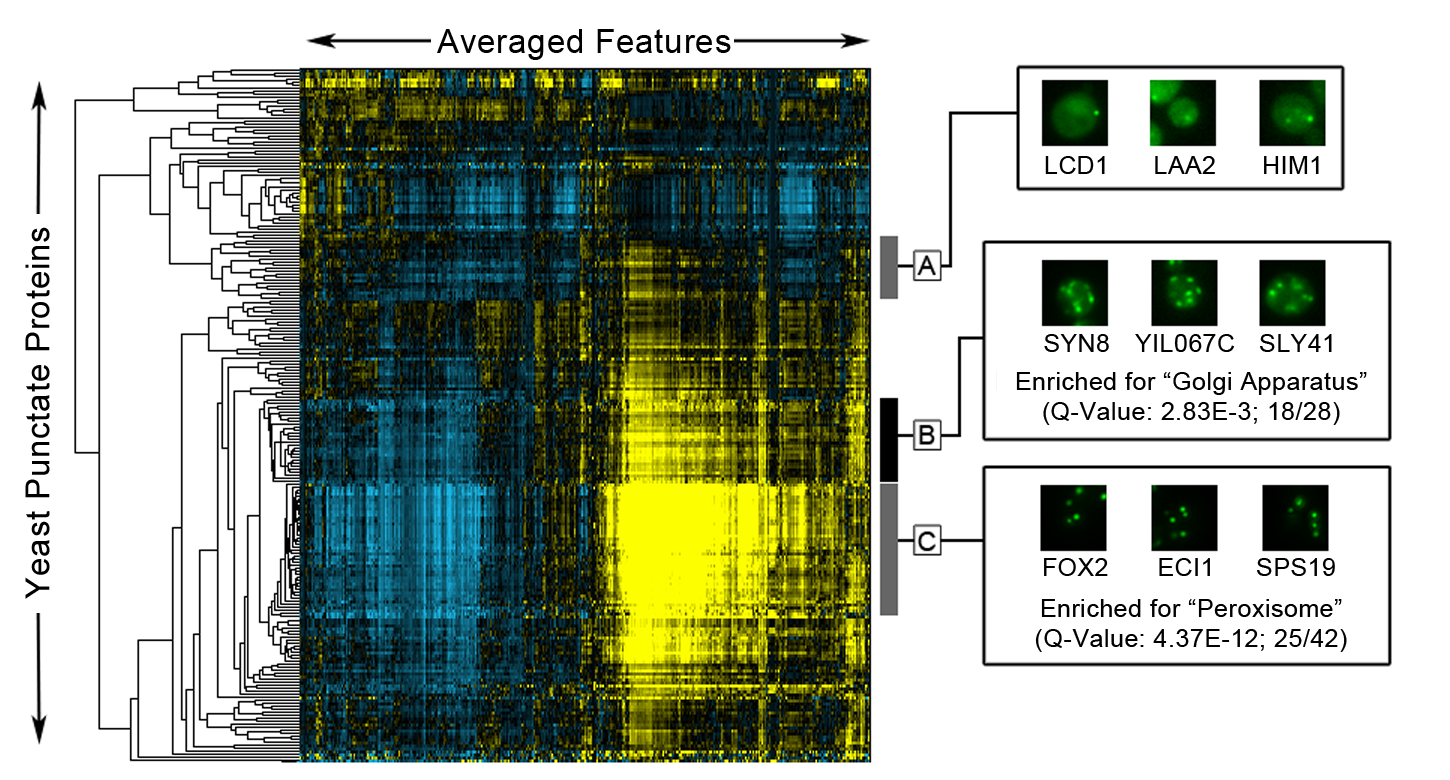

Supplement: S5 Fig — We visualize features as a heat map, where positive values are colored yellow and negative values are colored blue, with the intensity of the color corresponding to magnitude. Columns in this heat map are features, while rows are proteins. Features have been mean-centered and normalized using all proteins in the dataset. We show three clusters (black and grey bars on the right), and crops of three representative images of the proteins within each of the clusters. For image crops, we show the protein channel only in green. For clusters B and C, we show the GO enrichment of the clusters relative to all punctate proteins; we list the q-value (the FDR-corrected p-value) and the number of proteins in the cluster with that annotation relative to the full size of the cluster. (TIF) [file pcbi.1007348.s005.tif]
