# Supplementary material for: Learning unsupervised feature representations for single cell microscopy images with paired cell inpainting
Source: PLoS Comput Biol. 2019 Sep 3;15(9):e1007348. doi: 10.1371/journal.pcbi.1007348 (PMC6743779; doi:10.1371/journal.pcbi.1007348)
Supplement: S1 Table — Classification accuracies for single yeast cell localization classes using a kNN classifier on our test set of 30,889 labeled single cells, using various feature representations. We report the overall accuracy as the balanced accuracy of all classes. (DOCX) [file pcbi.1007348.s006.docx]

**Supplementary Table S1. Classification accuracies for feature sets with various parameterizations of *k***

| **Feature Set** | **Number of Neighbors (*k*)** | | | | |
| --- | --- | --- | --- | --- | --- |
|  | **1** | **5** | **11** | **25** | **50** |
| Designed Features | 57.16 | 61.06 | 62.04 | 61.51 | 59.99 |
| CellProfiler | 61.10 | 65.52 | 68.15 | 67.54 | 66.06 |
| Transfer Learning (VGG16) | 64.70 | 68.89 | 69.33 | 67.43 | 65.02 |
| Autoencoder | 35.31 | 40.32 | 42.50 | 42.50 | 47.19 |
| Paired Cell  Inpainting (Conv4) | 85.51 | 87.89 | 87.98 | 87.20 | 85.80 |
| Supervised | 90.09 | 92.25 | 92.44 | 92.33 | 92.16 |
