# Supplementary material for: Learning unsupervised feature representations for single cell microscopy images with paired cell inpainting
Source: PLoS Comput Biol. 2019 Sep 3;15(9):e1007348. doi: 10.1371/journal.pcbi.1007348 (PMC6743779; doi:10.1371/journal.pcbi.1007348)
Supplement: S2 Table — Classification accuracies for single yeast cell localization classes using logistic regression and random forest classifiers on our dataset of 30,889 labeled single cells, built on various feature representations. We also report accuracy of the classifier end-to-end for the fully-supervised CNN in the last row of the table. Metrics are reported as the average balanced accuracy on the test sets under 5-fold cross-validation. We implemented all classifiers in Python using the scikit-learn package. For our logistic regression classifiers, we used a L1 penalty with a balanced class weight. For our random forest classifiers, we used 500 trees with 20% of the features used to determine best splits, and a balanced class weight. (DOCX) [file pcbi.1007348.s007.docx]

**Supplementary Table S2. Classification accuracies for feature sets with logistic regression and random forest classifiers**

| **Feature Set** | **Accuracy  (Logistic Regression)** | **Accuracy (Random Forest)** |
| --- | --- | --- |
| Designed Features | 69.17 | 69.80 |
| CellProfiler | 80.00 | 78.17 |
| Transfer Learning (VGG16) | 83.77 | 72.29 |
| Autoencoder | 63.33 | 56.41 |
| Paired Cell  Inpainting (Conv4) | 91.34 | 87.49 |
| Supervised | 94.05 | 92.94 |
| Supervised **(End-to-End)** | 94.02 | |
